# *Torenia concolor* Lindley var. *formosana* Yamazaki extracts improve inflammatory response and lipid accumulation *via* PPARs activation

**DOI:** 10.1051/bmdcn/2017070318

**Published:** 2017-08-25

**Authors:** Yu-Chia Liang, Jun-Cheng Hu, Pei-Ying Li, Guan-Jhong Huang, Yueh-hsiung Kuo, Che-Yi Chao

**Affiliations:** 1 Department of Chinese Pharmaceutical Sciences and Chinese Medicine Resources, College of Chinese Medicine, China Medical University Taichung 404 Taiwan; 2 Department of Health and Nutrition Biotechnology, Asia University Taichung 413 Taiwan; 3 School of Pharmacy, College of Pharmacy, China Medical University Taichung 404 Taiwan; 4 Department of Biotechnology, Asia University Taichung 413 Taiwan; 5 Department of Medical Research, China Medical University Hospital, China Medical University Taichung 404 Taiwan

**Keywords:** *Torenia concolor* Lindley var. *formosana* Yamazaki, Anti-inflammation, Lipid metabolism, PPARs

## Abstract

Background/Introduction: At present, human diet is replete with sugar and fat. Abnormal metabolism and hyperglycemia or hyperlipidemia in the body induces the development of an overactive and continuous inflammatory response, resulting in obesity and metabolic syndromes, including hypertension, hyperlipidemia, and insulin resistance. *Torenia concolor* Lindley var. *formosana* Yamazaki (TC), a perennial creeping herbaceous plant, is a traditional Chinese medicinal herb widely used for the treatment of heat stroke, aching muscles and bones, cold, dysentery, and ambustion.

Purpose: This study evaluated the influence of TC on inflammation responses and lipid metabolism. Methods: In this study, ground TC powder was extracted with 95% ethanol. The ethanol was removed by vacuum concentration, and the resulting extract was further extracted with a number of solvents of different polarity to produce four final extracts: an ethanol extract (TCEE), an ethyl acetate extract (TCEAE), an n-butanol extract (TCBUE), and a water extract (TCWE). The anti-inflammatory efficacy of the extracts and their capability for lipid metabolism regulation was then explored.

Results: TCEE, TCEAE, and TCBUE exhibited good anti-inflammatory efficacy; TCEAE also simultaneously regulated lipid metabolism. In RAW264.7 cells, these three extracts suppressed the expression of iNOS and IL-6 *via* the signaling pathway activation of the transcription factor peroxisome proliferator activated receptor γ (PPARγ) and thereby showed anti-inflammatory efficacy. In 3T3-L1 cells, these three extracts promoted lipid metabolism and reduced lipid accumulation through the activation of PPARα and the increased expression of adiponectin, thus demonstrating regulation of lipid metabolism.

Conclusion: These results indicate that TC possesses anti-inflammatory efficacy and can regulate lipid metabolism through the activation of transcription factor PPARs. We speculate that these nutraceutical effects are attributable to betulin, an active ingredient in this herbal medicine.

## 1. Introduction

Nowadays, the human diet commonly contains a high content of carbohydrates, fats, and excess calories. Naturally, too much caloric intake will result in obesity. Obesity is one of the most common global metabolic disorders. Data from the WHO demonstrate that more than 1.3 billion adults worldwide are overweight (body mass index: 25-30 kg/m^2^) and a further 600 million are obese (body mass index: ≥ 30 kg/m^2^). Obesity is often concomitant with other symptoms (*e.g*., hypertension, hyperlipidemia, and insulin resistance), which are together termed metabolic syndrome. Such metabolic abnormalities and long-term hyperglycemia or hyperlipidemia are likely to induce overactive and continuous inflammatory reactions in the body, which may result in many severe diseases, such as cardiovascular disease (Axen *et al.,* 2003; Nabel, 2003), arthritis, Alzheimer’s disease, type 2 diabetes mellitus, and cancer (Hanada and Yoshimura, 2002).

Many epidemiologic studies have demonstrated the high mortality rate of obese individuals, especially due to cardiovascular disease. In morbidly obese individuals, their adipocytes are likely to be insulin-resistant, which means that they are unable to receive insulin signals to metabolize glucose, proteins, and fatty acids. Furthermore, on the one hand, insulin-resistant adipocytes may secrete excessive chemokines such as monocyte chemoattractant protein-1 (MCP-1) to attract circulating macrophages to infiltrate adipose tissue. The macrophages are stimulated by adipocyte-secreted fatty acids, and this generates inflammatory reactions. On the other hand, the macrophages may secrete increased amounts of tumor necrosis factor-α (TNF-α) to induce the transformation of normal adipocytes to new insulin-resistance adipocytes, and this generates a greater degree of severe chronic inflammatory reactions and metabolic syndrome in obese individuals. If the macrophage inflammatory reactions induced by adipose tissue secretions are ameliorated, the insulin resistance of adipocytes will be reduced, and chronic inflammatory reactions will be prevented.

Inflammation reactions are closely related to the progression of tissue damage caused by numerous diseases. Therefore, studies probing the anti-inflammatory effects of various medicines and compounds are extremely crucial. Peroxisome proliferator activated receptor γ (PPARγ) is a ligand-activated transcription factor of the nuclear receptor superfamily that controls the expression of a variety of genes involved in fatty acid metabolism, adipogenesis, inflammation, and insulin sensitivity.

Searching for nutraceutical ingredients from natural products that can prevent inflammation and obesity is pivotal in preventing diseases and promoting health. Consequently, the market for nutraceutical foods is large. *Torenia concolor* Lindley var. *formosana* Yamazaki (TC), which belongs to the Scrophulariaceae family, is a plant native to Taiwan. As stated in Pen-tsao Kang-mu, TC is efficacious in ameliorating “lung fire”, resolving phlegm, dissipating stasis, detoxifying, and releasing heat. Moreover, some previous studies have indicated that TC has anti-inflammatory active ingredients, e.g., betulin and maslinic acid (Lin *et al.,* 2009). It is with this in mind that the present study explored the efficacy of several different TC extracts in the improvement of inflammation and lipid metabolism in two cell types and investigated the possible molecular mechanisms of their action.

## 2. Materials and Methods

### 2.1. Chemicals and Reagents

Oil red O, 3-isobutyl-1-methylxanthine (IBMX), insulin from bovine pancreas and lipopolysaccharide (LPS) were purchased from Sigma (St. Louis, MO, USA). Thiazolyl blue tetrazolium bromide (MTT) and dimethyl sulfoxide (DMSO) were purchased from Amersco (Cochran, Ohio, USA). Dexamethasone (DEX) was purchased from Alfa Aesar (Heysham, Lancashire, UK). Primary antibodies against PPARα, PPARγ, and adiponectin were purchased from Thermo (Grand Island, NY, USA). Primary antibodies against β-actin and IL-6 were purchased from Genetex (Alton Parkway, Irvine, USA) and a primary antibody against iNOS was purchased from Novus (Southpark Way, Littleton, USA). Secondary antibodies against goat anti-mouse IgG and goat antirabbit IgG were purchased from Jackson Immunoresearch (West Grove, PA, USA). Mammalian Protein Extraction Reagent for protein extraction was purchased from Thermo (Grand Island, NY, USA). The Bradford reagent for protein quantification was purchased from Bio Basic Inc. (Markham, Ontario, Canada). The Total RNA Extraction Miniprep System kit for RNA extraction was purchased from Viogene (Taipei, Taiwan).

### 2.2. Extraction methods

The TC was ground to a powder, which was extracted with 95% ethanol followed by a partition extraction. For the ethanol extraction, TC powder was first soaked in 95% ethanol for 3 days. The ethanol was removed by vacuum concentration to produce a concentrate, which was weighed and then degassed with bubbling nitrogen gas to remove the remaining ethanol residue. This process generated an ethanol-free, concentrated extract. One aliquot of the extract was re-dissolved with ddH_2_O and placed into a partition-extraction flask into which ethyl acetate and *n*-butanol were sequentially added, in the order of increasing solvent polarity, for partition extraction. The two resulting organic extracts were concentrated by vacuum concentration and referred to respectively as TCEAE, composed of TC and ethyl acetate, and TCBUE, composed of TC and *n*-butanol. The remaining, concentrated aqueous phase after the extraction was referred to as TCWE and was composed of TC and water. The other aliquot of the aforementioned ethanol-free, concentrated extract, which was not used in the partition extraction, was set aside and referred to as TCEE. The organic extracts TCEAE, TCBUE, and TCEE were re-dissolved in absolute ethanol for the generation of stock solutions, whereas the TCWE extract was mixed with PBS (Phosphate-buffered saline) to produce a fourth stock solution. All of the four stock solutions were stored at -20 °C prior to further use.

### 2.3. Cell Culture

The mouse macrophage cell line RAW 264.7 (BCRC No. 60001) was obtained from the Bioresources Collection and Research Center (BCRC) of the Food Industry Research and Development Institute (Hsinchu, Taiwan) and routinely cultured in Dulbecco’s Modified Eagle’s Medium (DMEM; GIBCO- BRL, Grand Island, NY, USA) supplemented with 10% fetal bovine serum (FBS; GIBCO-BRL) and antibiotics (100 U*/ml* penicillin and 100 μg*/ml* streptomycin; GIBCO-BRL) at 37 °C in a humidified atmosphere with 5% CO_2_. The cells were subcultured every 2-3 days, and the medium was changed every 2 days. To assess the effects of the extracts on cell cytotoxicity, RAW264.7 cells were seeded in a 96-well plate at 1 × 10^4^ cells/well. After 24 h, the cells were treated with different concentrations of the four extracts or vehicle, and the cytotoxicity of the extracts was analyzed.

The mouse fibroblast 3T3-L1 cells (BCRC No. 60159) were also obtained from BCRC and maintained in DMEM containing 10% calf serum (CS; GIBCO-BRL) and antibiotics (100 U/*ml* penicillin and 100 μg/*ml* streptomycin; GIBCO-BRL). The cells were seeded in a 6-well plate at 5 × 10^5^ cells/well and grown to 100% confluence. To induce adipocytic differentiation, 2 days after reaching confluence (Day 0), the cells were exposed to a culture medium containing 0.5 mM 3-isobutyl-methylxanthine (IBMX), 1 DEX, and 1 μg/*ml* insulin (Bovine) (the MDI hormonal cocktail) for 2 days (Day 2). This medium was replaced with a fresh complete medium containing insulin with 10% FBS, and the cells were incubated for a further 2 days (Day 4). Thereafter, until the cells were fully differentiated, a fresh medium with 10% FBS was supplied every other day.

### 2.4. Measurement of nitrite production

Nitrite oxide (NO) has a short half-life and is a stable oxide metabolite; hence, the measurement of nitrite content would provide information regarding NO production. In this study, the TC cells were trypsinized, diluted to a concentration of 4 × 10^5^ cells/*ml* with culture medium, and then 100 μL of cell solution was inoculated into a 96-well plate. The plates were placed in an incubator filled with 5% CO_2_ at 37 °C for 18 h, after which the initial culture medium was removed from the plates, and they were then treated jointly with the samples and LPS and placed in an incubator (5% CO_2_, 37 °C) for 18 h. Subsequently, 100 μL of the supernatant was removed from each well by a pipette and transferred to a different 96-well plate. After the addition of Griess reagent (100 μL), the resulting solution was allowed to stand in the dark for 10 min before the absorbance of the solution at a wavelength of 540 nm was measured with an ELISA plate reader. The solution’s absorbance was compared with an absorbance calibration curve to determine its nitrite content.

### 2.5. Oil red O stain

Oil red O is a diazo dye, non-polar and liposoluble that stains the adipose droplets in adipocytes to form a red color that can be employed as a metric to evaluate adipose droplet content in adipocytes. In this study, a 0.35% solution of oil red O powder in isopropanol was mixed with deionized water in a water-to-stain ratio of 2:3 for use. After the completion of cell differentiation at different sample concentrations in 12-well plates, the plates were rinsed with PBS until the sample color was washed off, following which 300 μL of formaldehyde (10% solution in PBS) was added to each of the wells, allowed to stand for 1 h, and rinsed with deionized water by using pipettes until the formaldehyde odor had completely dissipated. Subsequently, 1 *ml* of 60% isopropanol was added to each well and allowed to stand for 2 min to ensure the full evaporation of the water. The oil red O stain was added to the wells, left to stand for 30 min, and then rinsed off with deionized water. Water was added to cover the cells and the cells were photographed, after which the wells were air-dried. Then, 1 *ml* of 60% isopropanol was added to each well, and each plate was shaken horizontally on a shaker until the red adipocytes were dissolved to form a solution. The absorbance of the solution was measured at the wavelength of 510 nm by an ELISA plate reader.

### 2.6. Protein extraction

The cell solutions were diluted to a concentration of 4 × 10^5^ cells/ *ml*, inoculated in 6-cm^2^ culture dishes, and placed in an incubator filled with 5% CO_2_ at 37 °C for 18 h. After removal of the culture medium, a culture medium containing LPS and the samples was added to the dishes, which were incubated in the incubator for a further 24 h, rinsed with PBS, and then the cells were collected on ice with a spatula. The collected cells were centrifuged at 4, 500 rpm for 5 min at 4 °C. After removal of the supernatant, 100 μL of cell lysis agent (M-PER®) was added to the centrifuge tube, removed, and re-added several times with a syringe. Subsequently, the tube was centrifuged at 1, 000 rpm for 15 min at 4 °C. The supernatant contained the original cellular proteins and was stored at -20 °C prior to use.

### 2.7. Protein quantification

A solution of bovine serum albumin was used to generate a calibration curve and a portion of the above extracted protein solution was diluted with ddH_2_O in order to ensure the protein concentration was within the dynamic range of the calibration curve. A 10-μL aliquot was transferred to a 96-well plate mixed Bradford reagent. The resulting mixture was allowed to stand for 2-3 min at room temperature. Subsequently, the absorbance of the solution was measured at a wavelength of 595 nm and used to evaluate the protein concentration based on the pre-determined calibration curve.

### 2.8. Western blotting

Proteins were resolved by SDS-polyacrylamide gel electrophoresis and then transferred to polyvinyl difluoride membranes. The blot membranes were blocked with 4% non-fat milk for 1 h at room temperature, followed by incubation with primary antibodies at 4 °C overnight. After washing three times, the blots were incubated with anti-rabbit or anti-mouse HRP-conjugated secondary antibodies for 1 h at room temperature. Finally, the blots were visualized by enhanced chemiluminescence using a Fujifilm LAS-3000 chemiluminescence detection system (Fujifilm; Tokyo, Japan).

### 2.9. Quantification software

AlphaEaseFC 4.0 imaging software was used to analyze the staining intensities of the western blots. The intensity of background staining was subtracted and the intensity of the target protein band was divided by the intensity of the control band to produce the quantitative data.

### 2.10. Statistical analysis

Each experimental data set was expressed as the mean ± standard deviation (mean ± sd). When the data were confirmed to follow a normal distribution, the data sets were subjected to one-way ANOVA and Duncan’s Multiple Range Test to determine whether there was a statistically significant difference between two independent sample groups (P < 0.05). All statistical analyses were conducted with Statistical Product and Service Solutions (SPSS) software version 18.0.

## 3. Results

### 3.1. The effect of the TC extracts on the survival rate and nitrite production in the RAW264.7 cells

RAW264.7 macrophages were treated with 100 ng/*ml* of LPS to induce inflammatory reactions and then treated with TC extracts. As shown in Table 1, under conditions that did not affect the cellular survival rate, this study analyzed the produced content of nitrite, an inflammatory mediator. It was found that 150 μg/*ml* of TCEE, 150 μg/*ml* of TCEAE, 250 μg/*ml* of TCBUE, and 400 μg/*ml* of TCWE all showed a significant inhibitory effect on nitrite production.

**Table 1:** The effects of the TC extracts on the nitrite production and cell survival rate in the RAW264.7

Treatment	Cell viability (%)	Nitrite production (μM)	Nitrite / Cell viability
Vehicle	100 ± 1.24^d^	250 ± 0.16^a^	33.28 ± 0.41^a^
LPS + Vehicle	101.54 ± 4.39^d^	16.96 ± 0.26^h^	98.63 ± 416^f^
LPS + TCEE 150μg/*ml*	111.89 ± 1.89^e^	7.40 ± 0.26^c^	59.63 ± 2.93^b^
LPS + TCEAE 150 μg/*ml*	102.00 ± 1.95^d^	8.46 ± 0.26^d^	45.44 ± 1.31^d^
LPS + TCBUE 250 μg/*ml*	119.78 ± 1.21^e^	5.71 ± 1.04^b^	50.55 ± 4.12^c^
LPS + TCWE 400 μg/*ml*	101.01 ± 1.59^d^	9.88 ± 0.46^e^	66.72 ± 3.92^e^
LPS + Dexamethasone 1 μM	110.20 ± 5.26^e^	5.41 ± 0.20^b^	42.43 ± 2.09^b^

Values are the mean ± SD (n = 5) and were analyzed using one-way ANOVA followed by Duncan’s new multiple range test. Dexamethasone (1 μM) is the positive control and cells were treated with 100 ng/*ml* LPS to induce inflammation.

### 3.2. The effect of the TC extracts on the inflammation-related protein expression in the RAW264.7 cells

As shown in Fig. 2, TCEE and TCEAE significantly reduced the expression level of iNOS protein, showing markedly greater inhibition than the positive control group (DEX) (P < 0.05). The inhibitory efficacies of TCBUE and TCWE were lower than those of TCEE or TCEAE, but were similar to the positive control group *(P* > 0.05). This study thus identified that, of the different TC extracts, TCEE and TCEAE exerted the strongest inhibitory effect on the expression of iNOS protein.

**Fig.1:**
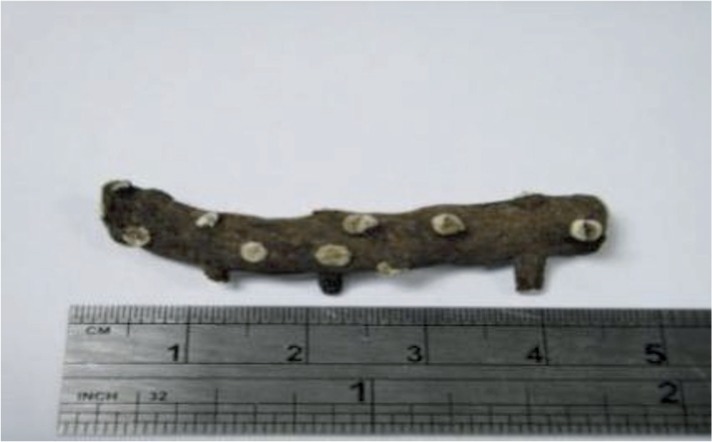
An experimental sample of TC

**Fig. 2:**
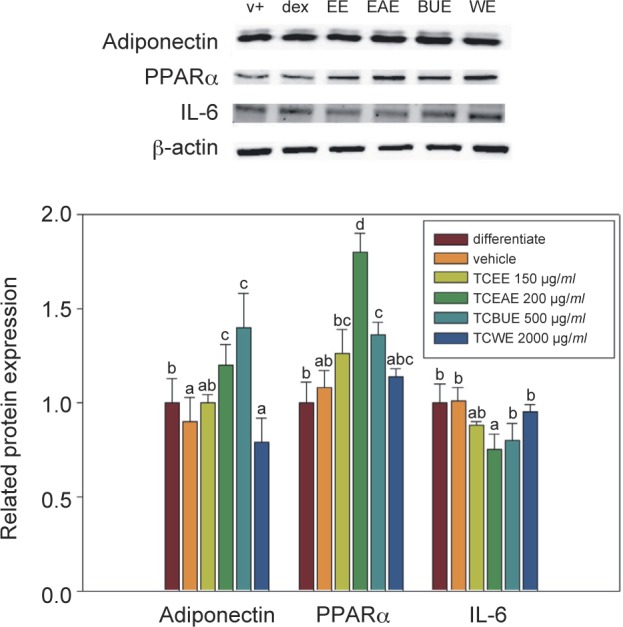
The effects of TCEE, TCEAE, TCBUE, and TCWE on the inflammatory-related protein expression in the RAW264.7 macrophages. Values are the mean ± SD (n = 3) and were analyzed using one-way ANOVA followed by Duncan’s new multiple range test. Bars not sharing a common letter are significantly different from each other (*P* < 0.05).

TCEE, TCEAE, and TCBUE significantly increased the expression level of the PPARγ protein. These increases were significantly different from what was observed in the blank group, the positive control group, and the negative control group *(P* < 0.05), while TCWE had no significant effect on the expression. This study found that, of the different TC extracts, TCEAE and TCBUE were able to significantly increase PPARγ protein expression.

TCEE and TCEAE both significantly reduced the protein expression level of the pro-inflammatory cytokine IL-6, with the observed inhibition being greater than that seen in the positive control group. Of the TC extracts, TCEAE was most effective inhibitor while TCBUE and TCWE showed the least effective inhibition. Thus, this study found that, of the different TC extracts, TCEE and TCEAE exerted the strongest inhibitory effect on the expression of IL-6 protein.

### 3.3. The effect of the TC extracts on the expression of lipid metabolism-related proteins in the 3T3- L1 cells

**Fig. 3:**
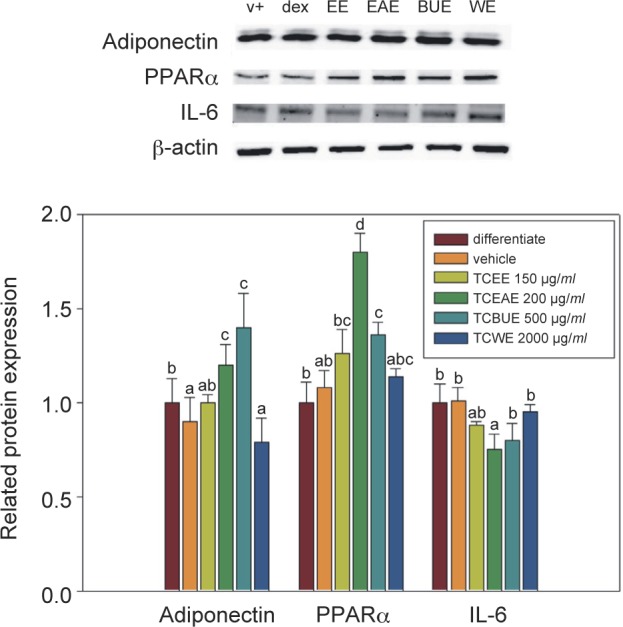
The effects of TCEE, TCEAE, TCBUE, and TCWE on the adipogenic-related protein expression in the 3T3- L1 adipocytes. Values are the mean ± SD (n = 3) and were analyzed using one-way ANOVA followed by Duncan’s new multiple range test. Bars not sharing a common letter are significantly different from each other (P < 0.05).

As shown in Fig. 3, TCEAE and TCBUE promoted the expression of adiponectin, and it ==they were not significantly different from the level in the differentiation group (*P* > 0.05). However, TCEE and TCWE reduced the expression level of adiponectin, with them not being significantly different from that of the vehicle group (*P* > 0.05). TCEAE and TCBUE promoted the level of adiponectin protein expression to 128% and 141% of that in the differentiation group, respectively. This study found that of the different TC extracts, TCEAE and TCBUE exerted the strongest effects on the promotion of adiponectin.

TCEE, TCEAE, and TCBUE significantly increased the expression level of the PPARα protein. TCEAE and TCBUE were the most efficient and significantly different from the differentiation group and the vehicle group (P < 0.05), whereas TCWE produced no significant effect on the PPARα protein expression. This study found that, of the different TC extracts, TCEAE had the most significant effect on increasing the expression level of the PPARα protein. TCEE and TCEAE inhibited IL-6 protein expression, but this inhibition was not significantly different from the differentiation group (*P* > 0.05). Meanwhile, the inhibitory effects of TCBUE and TCWE were even less significant. TCEE and TCEAE had inhibition rates of 16% and 23% compared with those in the differentiation group, respectively. The present study found that, of the different TC extracts, TCEE and TCEAE exerted the strongest inhibitory effect on the protein expression of pro-inflammatory cytokine IL-6.

### 3.4. The effect of the TC extracts on the lipid generation in the 3T3-L1 cells

**Fig. 4:**
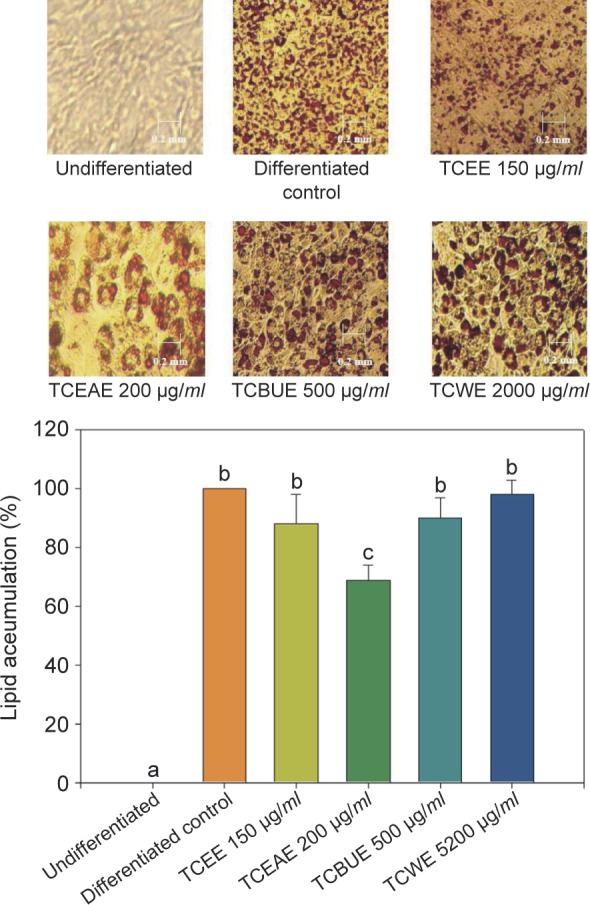
The effects of TCEE, TCEAE, TCBUE, and TCWE on the lipid accumulation in the 3T3-L1 adipocytes. Values are the mean ± SD (n = 3) and were analyzed using one-way ANOVA followed by Duncan’s new multiple range test. Bars not sharing a common letter are significantly different from each other (*P* < 0.05).

As shown in Fig. 4, 5 days after cell differentiation, 3T3-L1 preadipocytes were treated with TCEE, TCEAE, TCBUE, or TCWE at different concentrations for 72 h. The results of the MTT assay suggested that no cytotoxicity was observed at 150 μg/*ml* TCEE, 200 μg*/ml* TCEAE, 500 μg/*ml* TCBUE, or 2000 μg/*ml* TCWE. Therefore, these concentrations of TC extracts were used to treat the cells in the subsequent experiments and observe the effects on the generation of adipose droplets. Lipid differentiation was induced by a differentiation agent in 3T3-L1 pre-adipocytes and then the pre-adipocytes were treated with the TC extracts or their active ingredients. The generation of adipose droplets was observed under conditions that did not affect the cellular survival rate. The results showed that 200 μg/*ml* TCEAE exerted a significant inhibitory effect on the production of adipose droplets (*P* < 0.05), with an inhibition rate of 29.3%; no other TC extracts resulted in a statistically significant effect. Of the active ingredients, 100 μM betulin significantly inhibited the generation of adipose droplets (P < 0.05), with an inhibitory rate of 35.3%.

**Fig. 5:**
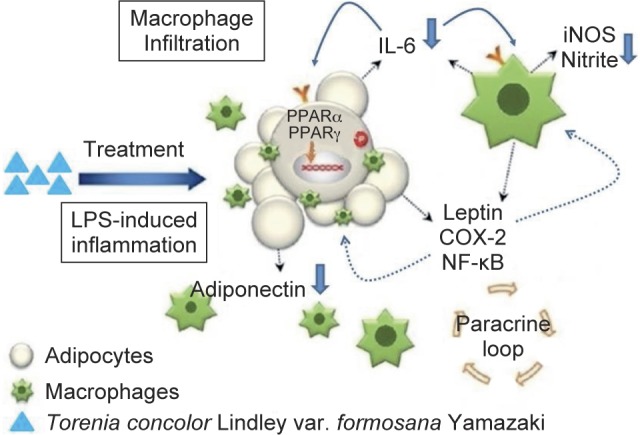
A suggested mechanism of TC for anti-inflammation and anti-lipid accumulation.

## 4. Discussion

### 4.1. The effect of the TC extracts on the inflammatory mediator production in the RAW264.7 cells

After the stimulation of macrophages by LPS, macrophage-induced inflammation will cause the acetylation of pro-inflam matory cytokines (Cooper *et al.,* 2010), and this exacerbates inflammation and leads to substantial NO production. Through metabolic processes, NO can be converted to nitrite, which plays a pivotal role in the course of inflammation. Nitrite can induce the production of other inflammatory substances, all of which cooperate to induce inflammatory reactions in the body. Studies have suggested that phenolic compounds, flavonoid compounds, and triterpenoids may inhibit LPS-induced inflammatory reactions in RAW264.7 murine macrophages (Chen *et al.,* 2011). The experimental results of this study indicated that TCEE, TCEAE, and TCBUE had significant inhibitory effects on nitrite production; in particular, TCEE had the most significant effect. At a concentration of 100 μg/*ml*, the inhibitory effect was satisfactory. However, when the concentration was increased to 150 μg/*ml*, the inhibitory effect on nitrite production was similar to that of DEX, an established anti-inflammatory drug. These results suggest that the anti-inflammatory mechanism of TC might occur through the L-arginine- NO pathway.

### 4.2. The effect of the TC extracts on the inflammation-related protein expression in the RAW264.7 cells

Both iNOS and IL-6 are important proteins in the course of inflammation. NOS (Nitric oxide synthase) can be classified into three types: neuronal nitric oxide synthase (nNOS), endothelial nitric oxide (eNOS), and inducible nitric oxide synthase (iNOS). iNOS exists mostly in macrophages, lymphocytes, and vascular smooth muscle cells. When those cells are stimulated, a high level of iNOS occurs, leading to a high production of NO (Nathan and Xie, 1994; Thiemermann, 1994). IL-6, meanwhile, exerts a pivotal role in the regulation of many immune functions, such as macrophage activation, B-cell development, inflammatory reactions, hematopoietic functions, and acute reactions. Therefore, an excess of IL-6 is usually associated with some diseases that arise from immune overreaction, such as rheumatoid arthritis and Crohn’s disease. IL-6 is a type of pro-inflammatory cytokines, which plays a pivotal role in acute inflammatory reactions and increases vascular permeability to elicit inflammatory reactions with symptoms such as red swelling, heat, and pain. It is the main regulator of continual stimulation and activation of macrophages for the secretion of more pro-inflammatory cytokines (Nathan, 1987). Activated PPARγ can inhibit the expression of the nuclear transcription factor NF-κB in the macrophages, reduce the activity of iNOS, and inhibit the production of IL-6 (Peng *et al.,* 2005; Jiang *et al.,* 1998). The TC extracts (TCEE, TCEAE, and TCBUE) all significantly inhibited the expression of the iNOS protein and subsequently inhibited the production of nitrite; similarly, they had a significant inhibitory effect on the expression of IL-6 and all significantly activated PPARγ protein expression. Given all the above findings and reports, we can speculate that TCEE, TCEAE, and TCBUE showed anti-inflammatory efficacy by inhibiting the expression of iNOS and the IL-6 protein through the signaling pathways of activated transcription factor PPARγ.

### 4.3. The effect of the TC extracts on the lipid generation in the 3T3-L1 cells

3T3-L1 pre-adipocytes begin to accumulate lipids after differentiation induced by IBMX. Studies have suggested that maslinic acid, an active triterpenoid in TC, could activate the Akt/PKB signaling pathway to promote insulin sensitivity and reduce lipid accumulation, as shown in experiments with 3T3- L1 adipocytes (Castellano *et al.,* 2013). However, some studies have found that stigmasterol, an active ingredient in steroids, could prevent ovarian, prostate, breast, and colon cancers, and also exert the potent nutraceutical effects of antioxidation and blood glucose decrease (Panda *et al.,* 2009). The results of this present study indicated that TCEAE had the best inhibitory effect on lipid generation, a fact which compelled us to speculate that the active ingredients of triterpenoids and steroids, such as betulin and maslinic acid, enabled TCEAE to regulate the lipid metabolism and decrease lipid production. However, a study involving further purification, isolation, and analysis is needed in order to identify and confirm these active ingredients.

### 4.4. The effects of the TC extracts on the expression of lipid metabolism-related proteins in the 3T3- L1 cells

Adiponectin, an adipocytokine that promotes insulin sensitivity, is secreted only by differentiated adipose tissues, and three types of secretions can occur: high, medium, and low molecular weight (Waki *et al.,* 2003; Tsao *et al.,* 2003). Of the three types, high-molecular-weight adiponectin has the highest metabolic activity, promoting insulin sensitivity and thereby alleviating the symptoms of diabetes mellitus (Pajvani *et al.,* 2004; Wang *et al.,* 2008; Yamauchi *et al*.,2013). Also, its blood concentration is lower in obese individuals than in other individuals (Arita *et al.,* 1999). IL-6 is a pro-inflammatory protein, but as 30% of IL-6 in the body is secreted by adipocytes, it is also an adipokine. The mechanism by which IL-6 influences insulin sensitivity is thought to include the following steps: IL-6 is produced by peritoneal fat and can directly enter and stimulate the liver to cause triglyceride secretion (Nonogaki *et al.,* 1995), affect insulin signal transduction, and influence insulin-induced glycogen production in hepatocytes (Senn *et al.,* 2002), the latter of which thereby reduces insulin sensitivity and leads to hyperglycemia (Tsigos *et al.,* 1997). Many disruptions in the regulation of lipid metabolism belong to the downstream genes of PPARα, *e.g.,* lipoproteins on and in the vascular endothelium, and apolipoprotein A-I (ApoA-I) and apolipoprotein A- II (ApoA-II) on the lipoprotein lipase. PPARα can promote lipoprotein lipase expression, causing lipoproteins to release fatty acids, helping them to enter tissues, and thereby facilitating their entrance into hepatocytes. Furthermore, PPARα was shown to regulate the expression of adipogenic-related genes and promote lipolysis in hepatocytes, reducing the fatty acids used for triglyceride production in the liver and thus decreasing the lipid concentration in blood (Schoonjans *et al.,* 1996). In the present study, TCEAE and TCBUE increased the levels of adiponectin, whereas TCEE and TCEAE inhibited the expression of the IL-6 protein; furthermore, TCEAE had a significant effect on the activation of the PPARα protein expression. Therefore, we speculated that the anti-lipid accumulation effect of TCEAE may have been achieved *via* the activation of PPARα and the subsequent increase in adiponectin expression, which in turn promoted lipid metabolism to reduce triglyceride production and enhance insulin sensitivity. Thus, the expression of IL-6 was inhibited and the regulation of lipid metabolism was achieved.

Obesity-related chronic inflammation partially arises from increased macrophage infiltration to adipose tissue. Those macrophages can release pro-inflammatory factors that influence adjacent adipocytes *via* paracrine and induce insulin resistance (Grimble, 2002, Cinti *et al.,* 2005, Hirasaka *et al.,* 2007). The present study found that the TCEE and TCEAE extracts had the strongest inhibitory effect on lipid production, and we speculated that botulin, maslinic acid, and stigmasterol, the active ingredients of triterpenoids and steroids in TC, enabled TC to regulate insulin resistance. Pro-inflammatory cytokines can increase IL-6 production, reduce adiponectin secretion, and influence insulin sensitivity, and these factors all create conditions suited to the development of metabolic syndrome. The regulation of inflammatory factors *(e.g.,* NF-κB, TNF-α, IL-6, COX-2, and iNOS) and adipogenic related factors (e.g., IL-1β, MCP- 1, IL-6, CRP, PAI-1, PPARα and adiponectin) can lead to the suppression of inflammatory reactions (Hsu *et al.,* 2013). In addition to the induction of inflammatory factors in RAW264.7 macrophages, LPS also induces inflammatory factors in 3T3-L1 adipocytes, e.g., the activation of the NF-κB transduction pathway and the pro-inflammatory cytokines *(e.g.,* IL-6, TNF-α and IL-10) to affect insulin sensitivity (Chirumbolo *et al.,* 2014). TCEAE inhibited IL-6 production, but it also substantially activated the expression of the PPARα protein. PPARs are regarded as therapeutic targets for cardiovascular disease: the activation of these not only directly regulates the genes of vascular and inflammatory cells involved in atherosclerosis, but also indirectly promotes glucose utilization and serum lipid profiles. Prior research in our laboratory found that TC extracts could activate PPARα and PPARγ (data not shown). Therefore, we speculated that TCEAE could improve macrophage infiltration-induced insulin resistance through the activation of PPARα to reduce the production of pro-inflammatory factors in the macrophages, inhibit the generation of pro-inflammatory cytokine IL-6, activate PPARα and increase the expression of adiponectin, which all could lead to the regulation of lipid metabolism and the amelioration of insulin sensitivity.

## 5. Conclusions

TCEE, TCEAE, and TCBUE inhibited the production of nitrite (an inflammatory mediator) *via* the inhibition of iNOS protein expression in RAW264.7 cells and inhibited the expression of pro- inflammatory cytokine IL-6 *via* the activation of transcription factor PPARγ and the increase in its expression, these three extracts demonstrated good anti-inflammation efficacy. TCEAE reduced the production of adipose droplets *via* an increase in adiponectin and an activation of PPARα protein expression in 3T3-L1 cells, thereby demonstrating a good capacity to alleviate lipid accumulation.

This study demonstrated the potential of TC in the development of nutraceutical foods to prevent inflammation and obesity. In the future, column chromatography may be employed to identify the composition of TCEAE; from this, the active ingredients responsible for the anti-inflammatory and anti-lipid accumulating effects may be isolated and purified for further animal experiments in which the nutraceutical effects of the active ingredients and their molecular mechanism could be even further explored.

## References

[1] Arita Y, Kihara S, Ouchi N, Takahashi M, Maeda K, Miyagawa J, *et al* Paradoxical decrease of an adipose- specific protein, adiponectin, in obesity. Biochem Biophys Res Commun. 1999, 257(1): 79–83. 10092513 10.1006/bbrc.1999.0255

[2] Axen KV, Dikeakos A, Sclafani A. High dietary fat promotes syndrome X in nonobese rats. J. Nutrition. 2003, 133(7):2244–2249. 12840187 10.1093/jn/133.7.2244

[3] Castellano JM, Guinda A, Delgado T, Rada M, Cayuela A. Biochemical Basis of the Antidiabetic Activity of Oleanolic Acid and Related Pentacyclic Triterpenes. Diabetes. 2013, 62(6):1791–1799. 23704520 10.2337/db12-1215 PMC3661625

[4] Chen HJ, Chung CP, Chiang W, Lin YL. Anti-inflammatory effects and chemical study of a flavonoid-enriched fraction from adlay bran. Food Chem. 2011, 126(4): 1741–1748. 25213953 10.1016/j.foodchem.2010.12.074

[5] Chirumbolo S, Franceschetti G, Zoico E, Bambace C, Cominacini L, Zamboni M. LPS response pattern of inflammatory adipokines in an in vitro 3T3–L1 murine adipocyte model. Inflamm. Res. 2014, 63: 495–507. 24526004 10.1007/s00011-014-0721-9

[6] Cinti S, MiTChell G, Barbatelli G, Murano I, Ceresi E, Faloia E, Wang S, Fortier M, Greenberg AS, Obin MS. Adipocyte death defines macrophage localization and function in adipose tissue of obese mice and humans. J. Lipid Res. 2005; 46(11): 2347–2355. 16150820 10.1194/jlr.M500294-JLR200

[7] Cooper ZA, Singh IS, Hasday JD. Febrile range temperature represses TNF-alpha gene expression in LPS-stimulated macrophages by selectively blocking recruitment of Sp1 to the TNF-alpha promoter. Cell Stress Chaperones. 2010, 15(5): 663–673. 10.1007/s12192-010-0179-9 PMC3006616 20221720

[8] Grimble RF. Inflammatory status and insulin resistance. Curr Opin Clin Nutr Metab Care. 2002, 5)5): 551–559. 12172480 10.1097/00075197-200209000-00015

[9] Hanada T, Yoshimura A. Regulation of cytokine signaling and inflammation. Cytokine Growth Factor Rev. 2002, 13(4–5): 413–421. 12220554 10.1016/s1359-6101(02)00026-6

[10] Hirasaka K, Kohno S, Goto J, Furochi H, Mawatari K, Harada N, *et al* Deficiency of Cbl-b gene enhances infiltration and activation of macrophages in adipose tissue and causes peripheral insulin resistance in mice. Diabetes. 2007, 56(10): 2511–2522. 17601987 10.2337/db06-1768

[11] Hsu CL, Lin YJ, Ho CT, Yen GC. The Inhibitory Effect of Pterostilbene on Inflammatory Responses during the Interaction of 3T3-L1 Adipocytes and RAW 264.7 Macrophages. J Agric Food Chem. 2013, 61(3):602–610. 23268743 10.1021/jf304487v

[12] Jiang C, Ting AT, Seed B. PPAR-gamma agonists inhibit production of monocyte inflammatory cytokines. Nature. 1998, 391(6662): 82–86. 9422509 10.1038/34184

[13] Lin YC, Cheng HY, Huang TH, Huang HW, Lee YH, Peng WH. Analgesic and anti-inflammatory activities of Torenia concolor Lindley var. formosana Yamazaki and betulin in mice. Am J Chin Med. 2009, 37: 97–111. 19222115 10.1142/S0192415X09006606

[14] Nabel EG. Cardiovascular disease. N Engl J Med. 2003, 349(1): 60–72. 12840094 10.1056/NEJMra035098

[15] Nathan C, Xie QW. Nitric oxide synthases: roles, tolls, controls. Cell. 1994, 78(6): 915–918. 7522969 10.1016/0092-8674(94)90266-6

[16] Nathan CF. Secretory products of macrophages. J Clin Investig. 1987; 79(2): 319–323. 3543052 10.1172/JCI112815 PMC424063

[17] Nonogaki K, Fuller GM, Fuentes NL, Moser AH, Staprans I, Grunfeld C, Feingold KR. Interleukin-6 stimulates hepatic triglyceride secretion in rats. Endocrinol. 1995, 136(5): 2143–2149. 10.1210/endo.136.5.7720663 7720663

[18] Pajvani UB., Hawkins M, Combs TP, Rajala MW, Doebber T, Berger JP, Wagner JA, Wu M, Knopps A, Xiang AH, Utzschneider KM, Kahn SE, Olefsky JM, Buchanan T, Scherer PE. Complex distribution, not absolute amount of adiponectin, correlates with thiazoli-dinedione-mediated improvement in insulin sensitivity. Biol Chem. 2004, 279(13):12152–12162. 10.1074/jbc.M311113200 14699128

[19] Panda S, Jafri M, Kar A, Meheta BK. Thyroid inhibitory, antiperoxidative and hypoglycemic effects of stigmasterol isolated from Butea monosperma. Fitoterapia. 2009, 80(2): 123–126. 19105977 10.1016/j.fitote.2008.12.002

[20] Peng Y, Liu H, Liu F, Wang H, Liu Y, Duan S. Inhibitory effect of PPAR-gamma activator on IL-6 and mPGES protein expression in PBMC induced by homocysteine. Hemodial Int. 2005, 1: S15–S20. 10.1111/j.1542-4758.2005.01165.x 16223437

[21] Schoonjans K, Staels B, Auwerx J. The peroxisome proliferator activated receptors (PPARs) and their effects on lipid metabolism and adipocyte differentiation. BBA Lipid Lipid Metabol. 1996, 1302(2): 93–109. 10.1016/0005-2760(96)00066-5 8695669

[22] Senn JJ, Klover PJ, Nowak IA, Mooney RA. Interleukin-6 induces cellular insulin resistance in hepatocytes. Diabetes. 2002, 51(12):3391–3399. 12453891 10.2337/diabetes.51.12.3391

[23] Thiemermann C. The role of the L-arginine: nitric oxide pathway in circulatory shock. Adv Pharmacol. 1994, 28: 45–79. 7521665 10.1016/s1054-3589(08)60493-7

[24] Tsao TS, Tomas E, Murrey HE, Hug C, Lee DH, Ruderman NB, Heuser JE, Lodish HF. Role of disulfide bonds in Acrp30/adiponectin structure and signaling specificity. Different oligomers activate different signal transduction pathways. J Biol Chem. 2003, 278(50): 50810–50817. 14522956 10.1074/jbc.M309469200

[25] Tsigos C, Papanicolaou DA, Kyrou I, Defensor R, Mitsiadis CS, Chrousos GP. Dose- dependent effects of recombinant human interleukin-6 on glucose regulation. J. Clin. Endocrinol. Metabol. 1997, 82(12): 4167–4170. 10.1210/jcem.82.12.4422 9398733

[26] Waki H, Yamauchi T, Kamon J, Ito Y, Uchida S, Kita S, *et al* Impaired multimerization of human adiponectin mutants associated with diabetes. Molecular structure and multimer formation of adiponectin.. J Biol Chem. 2003, 78: 40352–40363. 10.1074/jbc.M300365200 12878598

[27] Wang Y, Lam KS, Yau MH, Xu A. Post-translational modifications of adiponectin: mechanisms and functional implications. Biochem J. 2008, 409(3): 623–633. 18177270 10.1042/BJ20071492

[28] Yamauchi T, Kadowaki T. Adiponectin receptor as a key player in healthy longevity and obesity-related diseases. Cell Metabol. 2013, 17(2): 185–196. 10.1016/j.cmet.2013.01.001 23352188

